# Back to basics: Precision while mixing total mixed rations and its impact on milking performance

**DOI:** 10.3168/jdsc.2023-0423

**Published:** 2023-11-17

**Authors:** Alex Bach

**Affiliations:** ^1^ Marlex Recerca i Educació, 08173 Barcelona, Spain; ^2^ Institució de Recerca i Estudis Avançats (ICREA), 08010 Barcelona, Spain

## Abstract

Details from every mixing load of total mixed ration (TMR) fed to ∼19,000 cows distributed in 92 pens from 21 farms, along with individual milk yield of each cow in every pen and farm, were collected from a feeding and management system (algoMilk; www.algomilk.com) between 2020 and 2022 on a daily basis to assess the impact of quality of mixing TMR on animal performance. Divergence between expected and actual amounts of ingredients mixed in every load was calculated and expressed as a percentage relative to expected amounts. Ingredients were classified as (1) energy grains (i.e., corn, wheat), (2) protein sources (i.e., soybean meal, canola meal), (3) hays (i.e., alfalfa hay), (4) grain silages (i.e., corn silage), (5) nongrain silages (i.e., alfalfa silage), (6) minerals (i.e., salt, sodium bicarbonate), and (7) straw (i.e., wheat straw). Milk yield was averaged within farm and pen on a weekly basis, and mixing divergences were also averaged by load or by ingredient type and week within pen and farm. The weekly standard deviation (SD) of mixing divergences was calculated for every pen and farm. The average divergence of the total amount of TMR prepared was 1.52 ± 0.017% (mean ± SD), which means that, in general, mixing errors were caused by adding an excess of one or more ingredients. Energy grains (1.20 ± 0.037%), grain silages (1.78 ± 0.023%), hays (2.29 ± 0.044%), and protein sources (1.82 ± 0.043%) were mixed in excessive amounts (mean ± SD), whereas nongrain silages (−1.5 ± 0.037%), molasses (−3.05 ± 0.067%), minerals (96.9 ± 0.084%), and straw (−0.6 ± 0.063%) were mixed (mean ± SD) in lower amounts than expected. Divergence in the total amount of TMR was weakly quadratically (concave) correlated (R^2^ = 0.04) with milk yield. Similarly, divergence in the amounts of grains, protein sources, hay, silages, minerals, and molasses mixed in the TMR wagon in relation to the formulated ration was also weakly and quadratically (concave) with milk yield. Minimizing mixing errors may have positive effects on milking performance.

## Highlights

• Producers mix greater total amounts of TMR than what would be dictated by the formulated ration.
• Grains, protein sources, hay, and grain silages are mixed in excessive amounts in relation to the formulated ration.
• Minerals, molasses, straw, and nongrain silages are mixed in deficient amounts in relation to the formulated ration.
• Divergences in total amount of feed or in individual ingredients are quadratically associated with milk yield.

## Graphical Abstract

**Figure:**
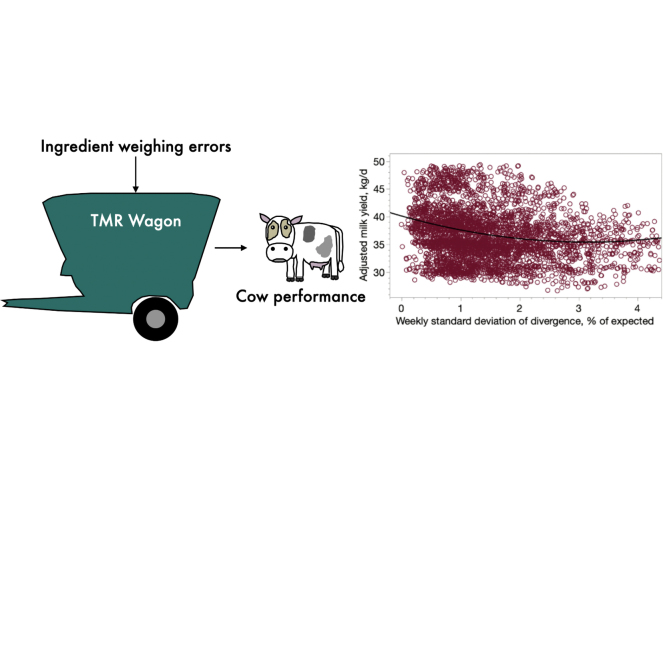
**Summary** Mixing total mixed rations (TMR) is an important and error-prone activity on dairy farms. Variations in TMR composition due to mixing errors may impair production and negatively affect health, as small deviations in nutrient supply may force cows to either mobilize their own nutrient reserves, if available, or reduce milk production or any other physiological function. Producers consistently overmix energy grains, grain silages, hays, and protein sources, whereas nongrain silages, molasses, minerals, and straw are added in lower amounts than expected. Divergence in the total amount of TMR was weakly but quadratically correlated with milk yield. Similarly, divergence in the amounts of grains, protein sources, hays, silages, minerals, and molasses mixed in the TMR wagon in relation to the formulated ration was also weakly and quadratically correlated with milk yield. Minimizing mixing errors may have positive effects on milking performance.

Since the introduction of the concept of a TMR in the late 1960s (McCoy et al., 1966), this feeding system has progressively become the standard for dairy cattle, and nowadays the vast majority of dairy herds across the globe that produce milk intensively feed cows using TMR. A relatively recent survey (USDA National Animal Health Monitoring System, 2014) reported that ∼90% of large herds (>500 cows/herd) feed a TMR. Preparing and feeding TMR requires some diligence to calculate the amounts of each ingredient that should be mixed daily as dictated by the formulated ration and the number of animals to feed (Schingoethe, 2017), and thus, the ration prepared by producers may not accurately reflect the recipe originally formulated by nutritionists. Nutrient delivery is essential to sustain milk production but also to cover all physiological needs of cows. Stone (2003) speculated that variations in TMR composition may impair production and negatively affect health. Indeed, small deviations in nutrient supply force cows to either mobilize their own nutrient reserves, if available, or reduce milk production or any other physiological function, such as reproduction. Previous studies in this field have mainly focused on assessing variation in nutrient and ingredient composition of TMR and divergences between expected and actual composition (Trillo et al., 2016; Carneiro et al., 2021; Cherney et al., 2021), with just a few studies (Rossow and Aly, 2013; Sova et al., 2014) looking at potential associations between mixing consistency and milking performance. Sova et al. (2014) reported a negative impact on milk production of the variation in the TMR content of long particles. However, even if cows would be fed a TMR with homogeneity that would limit sorting, the TMR delivered may still not match the ration formulated by the nutritionist or be inconsistent in composition between days. Herein, it is hypothesized that divergence in the amount of TMR or in the amount of some of its individual components between expected (theoretical ration) and actual may negatively affect milk production. The objective of this study was to assess the potential impact of quality of mixing TMR on milking performance of dairy cows.

No approval from an Animal Care Committee was sought for this study because it was conducted entirely on retrospective data collected from electronic records of the herds with cows that were kept under commercial rearing conditions and producers received no instructions to alter or modify their daily routines and management. Also, personal data relative to the persons manufacturing the rations were not part of the data collected from the farms. A dataset containing daily records between 2020 and mid-2023 of individual milk production of 18,926 cows along with their DIM from 21 herds (located in Italy, Portugal, Spain, and the Netherlands), the amounts of each ingredient of each ration offered to every pen (92 pens in total), and the theoretical ingredient composition of the ration for every pen and herd was extracted from a management and feeding system that integrates data from different sources and assists producers using artificial intelligence (algoMilk, www.algomilk.com, Spain). The weekly average and standard deviation (**SD**) of milk production within every pen and herd was calculated. Also, the difference between the total amount of feed that was delivered to every pen and the expected amount was expressed as a percentage: [total actual amount/total expected amount] × 100 on a daily basis (i.e., if the expected amount was 120 kg and the amount mixed was 125 kg, the divergence was considered to be 104.2%). Likewise, the divergence between the amount of every ingredient loaded in the TMR wagon and the expected (based on the theoretical ration) amount was determined daily for every pen. Again, this diversion was expressed as a percentage: [actual amount of ingredient *i*/expected amount of ingredient *i*] × 100. Then, as performed with milk yield data, the weekly average and SD of mixing divergences between actual and expected deliveries for every pen and herd was calculated. Table 1 provides descriptive statistics of the farms.

**Table 1:** Descriptive statistics of the 21 farms enrolled in the study

Item	Mean	Median	Minimum	Maximum
Number of lactating cows,/herd	543	473	206	1,115
Number of pens for lactating cows,/herd	4.3	4	1	8
Milk production, kg/cow·d	37.0	37.6	34.3	44.8
DIM, d	171	164	140	224
DM offered, kg/cow·d	25.4	25.5	14.7	36.3
Proportion of formulated rations prepared,^1^ %	102.1	102.0	70	134
Total TMR load, kg·pen/d	6,881	5,791	1,540	12,562
Average daily divergence TMR, kg·pen/d	91	67	−370	699
Average daily divergence TMR, %·pen/d	101.5 (0.03)*	101.2	96.4	106.6
Average daily divergence, %·pen/d				
Nongrain silages	98.5 (0.09)*	98.8	87.1	111.3
Grain silages	101.8 (0.04)*	101.2	86.7	119.7
Hays	102.3 (0.08)*	101.8	81.3	123.3
Straw	99.4 (0.15)*	99.7	79.0	122.3
Grains	101.2 (0.07)*	100.3	81.7	121.6
Protein sources	101.8 (0.12)*	100.6	90.0	118.9
Molasses	97.0 (0.22)*	96.4	79.6	117.3
Minerals	96.9 (0.04)*	96.5	79.6	117.3

1 Denotes the proportional increase or decrease of kilograms of ration per animal and day relative to the formulated ration. For example, if a ration was formulated for 24.5 kg/d of intake but cows were consuming 25.0 kg/d and producers were aiming at 2% refusals (thus preparing 25.5 kg·animal/d), then this value would be 104%.

* Denotes values differing from 100 at *P* < 0.05. Values within parentheses indicate SEM.

Ingredients across farms were classified in 8 categories: (1) grains (i.e., corn, wheat, sorghum), (2) protein sources (i.e., soybean meal, canola meal, sunflower meal), (3) hays (i.e., alfalfa hay, ryegrass hay), (4) grain silages (i.e., corn silage, oat silage), (5) nongrain silages (i.e., alfalfa silage, ryegrass silage), (6) minerals (i.e., salt, magnesium oxide), (7) molasses (i.e., molasses), and (8) straw (i.e., wheat straw). The weekly averages of divergences, expressed as a percentage of expected amounts for the total amount of TMR for every ingredient, were calculated and checked for normality. Next, to determine if these weekly averages of divergences in the total amount of TMR prepared or the corresponding ingredient types were consistently under- or oversupplied to the TMR mixer, they were tested against 100 with a *t*-test using JMP (version 17.0, SAS Institute Inc., Cary, NC). Also, the potential effect of weekly mixing divergences (expressed both as average of percent divergences or as their SD) on milk yield was assessed using JMP (version 17, SAS Institute Inc.) and a 2-degree polynomial mixed-effects model accounting for the random effects of farm and pen within farm, and the continuous (and fixed) effect of the weekly mixing divergences with weekly average DIM within pen and farm as a covariate. This model was run for divergences in the total amount of TMR prepared and also for individual ingredient types (using the categories described above). To determine if the obtained models were overly influenced by specific observations with an excessive leverage on the fit, the Cook's distance (Cook, 1979) was calculated for every point. Points with a Cook's distance greater than 4/number of observations would be considered excessively influential. For easier visual interpretation of data, an adjusted milk yield was calculated by adding the corresponding residual from the fitted model to each prediction of milk yield derived from the model (St-Pierre, 2001).

With the feeding software used in these farms, producers had a display in the TMR wagon indicating the expected amount of each ingredient (based on the formulated ration, the number of cows in each pen, and the number of rations, or level of refusals, they wanted to prepare every day for every pen), and the software recorded the actual amount added into the wagon; thus, divergences between expected and actual amounts were mainly due to weighing errors. Overall, producers consistently added excessive amounts (1.52 ± 0.017% surplus; mean ± SD) when preparing the TMR relative to what was expected based on the formulated ration and the number of animals to feed (Table 1). This observation is in agreement with previous reports (Trillo et al., 2016). There was a quadratic relationship with a concave shape (adjusted milk yield, kg/d = −2,771 (±254.6) + 55.36 (±4.995) × percent divergence − 0.273 (±0.0245) × percent divergence^2^; R^2^ = 0.04, *P* < 0.01; values within parentheses here and in the following equations denote SE) between weekly divergences of TMR mixed (relative to expected amounts) and average weekly milk yield (Figure 1A). No point had a Cook's distance >0.0009, and thus the fit was considered not be slanted by specific observations. Therefore, mixing greater or mixing lesser amounts of feed relative to what is expected has a negative impact on milk production. Similarly, the weekly SD of the divergences in the total amount of TMR prepared (also expressed as a percentage relative to expected amounts) was quadratically (adjusted milk yield, kg/d = 40.18 [±0.34] − 2.995 [±0.424] × weekly SD of divergence + 0.4577 [±0.110] × weekly SD of divergence^2^, R^2^ = 0.03, *P* < 0.01) correlated (with a convex shape) with milk yield (Figure 1B). Again, no point had a Cook's distance >0.0009. As the weekly SD of mixing divergences increased, milk production decreased, and the marginal decrease was more marked when weekly SD were <2%, with weekly SD >2% having small marginal impacts on milk yield (Figure 1B). Sova et al. (2014) reported a similar negative association, although in that case it was linear, not quadratic, between the coefficient of variation of dietary NE_L_ and milk yield. Nevertheless, these associations between TMR divergences and milk yield are very small; they explain <4% of the observed variation in milk yield. However, when attempting to optimize economic returns and milking performance in herds, this small variation may actually become important in the long run, especially when considering that, due to these uncertainties in the actual composition of the TMR offered, consultants often use safety margins when formulating rations that result in the provision of an excess of nutrients, which may lead to more expensive rations and an inefficient use of natural resources (Bach et al., 2020).

**Figure 1:**
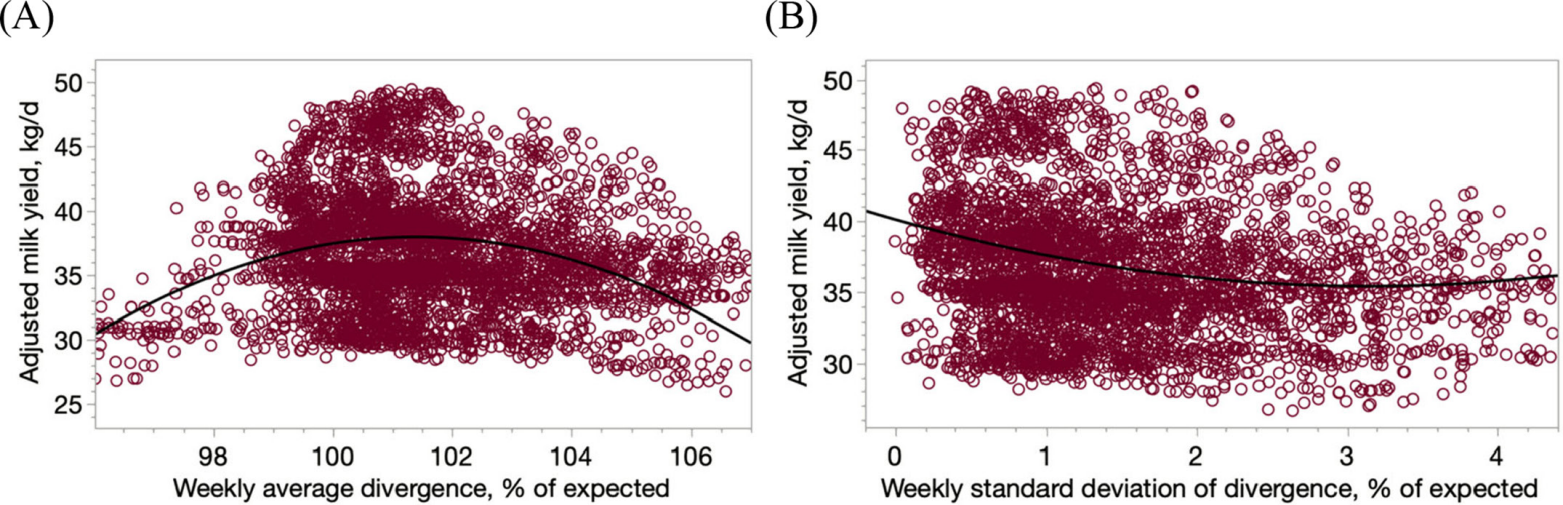
Effect of weekly mixing divergences expressed as a percentage relative to expected amounts (A; adjusted milk yield, kg/d = −2,771 + 55.36 × percent divergence − 0.273 × percent divergence^2^; R^2^ = 0.04, *P* < 0.01) or as the weekly SD relative to the expected amounts (B; adjusted milk yield, kg/d = 40.18 − 2.995 × weekly SD of divergence + 0.4577 × weekly SD of divergence^2^, R^2^ = 0.03, *P* < 0.01) in the TMR on milking performance.

Divergences in total amount of TMR mixed were not homogeneously distributed among ingredients and were likely a result of weighing errors of one or several ingredients, with some being underfed and others consistently overfed (Table 1). Sova et al. (2014) reported relatively large discrepancies in the nutrient content of prepared TMR with respect to the expected composition. These discrepancies could be due to errors in estimating nutrient content of the ingredients, mixing wrong amounts of ingredients in the TMR wagon, or both. In the current study, energy grains (1.20 ± 0.037%), grain silages (1.78 ± 0.023%), hays (2.29 ± 0.044%), and protein sources (1.82 ± 0.043%) were mixed in excessive amounts (mean ± SD), whereas nongrain silages (−1.5 ± 0.037%), molasses (−3.05 ± 0.067%), minerals (96.9 ± 0.084%), and straw (−0.6 ± 0.063%) were mixed (mean ± SD) in lower amounts than expected (Table 1). These results are in line with previous reports on mixing consistencies. Trillo et al. (2016) also reported that producers overfed corn silage, protein source, hay, and grains, and underfed liquids and minerals, relative to theoretical amounts that should be provided according to the formulated ration. Similarly, Moallem and Lifshitz (2020) reported that grains were consistently added in excessive amounts while mixing TMR. Nongrain silages are typically fairly wet and it is difficult to weigh the right amount and add it to the TMR wagon, but unfortunately, DM content of silages was not recorded in the current study. Another issue contributing to the mixing errors could be the total amount of silage that needs to be added to the TMR wagon. The average amount of nongrain silages added to each TMR was 843 kg, representing about 13% of the total TMR load. This relatively small amount could also be responsible for the large discrepancies between expected quantities according the formulated ration and actual deliveries to the wagon. Molasses, and other liquid ingredients, take a bit of time to load to the TMR wagon and it could be speculated that producers seem to not fully wait until the target amount is delivered. Similar to the argument made with nongrain silage, the relatively small amount of molasses added to the mixed could also be partially responsible for the large divergences. The divergences in the amounts of individual ingredient types added to the TMR wagon were associated with weekly average milk yield. For example, as shown in Figure 2, divergence in nongrain silages (adjusted milk yield, kg/d = −507.27 [±98.31] + 10.952 [±1.994] × weekly average divergence − 0.0551 [±0.010] × weekly average divergence^2^; R^2^ = 0.03, *P* < 0.01), grain silages (adjusted milk yield, kg/d = −106.97 [±67.21] − 3.142 [±1.296] × weekly average divergence − 0.0170 [±0.006] × weekly average divergence^2^; R^2^ = 0.02, *P* < 0.01), protein source (adjusted milk yield, kg/d = −62.74 [±42.7] + 1.99 [±0.82] × weekly average divergence − 0.01 [±0.004] × weekly average divergence^2^; R^2^ = 0.03, *P* < 0.01), and straw (adjusted milk yield, kg/d = −55.55 [±16.25] + 1.744 [±0.330] × weekly average divergence − 0.0084 [±0.0017] × weekly average divergence^2^; R^2^ = 0.04, *P* < 0.01) had quadratic (with concave shape) associations with milk yield. Cook's distance did not highlight a problematic data point in any of these regression fits. The decay in milk yield as mixing divergences in protein sources and grain silage increased was similar, with a more pronounced drop with divergences >100% (i.e., mixing greater amounts than expected) and almost no effect with divergences <100% (i.e., mixing lower amounts than expected). For the case of protein sources this could be related to the additional energy that cows would need to divert toward excreting the excess of N consumed (relative to the energy provided and their protein requirements). For the case of grain silages, Dhiman and Satter (1997) compared 3 levels of corn silage inclusion in the TMR and reported a curvilinear response, with an optimum when corn silage represented one-third of the dietary forage fraction, with proportions above or below resulting in lower milk yields. On the other hand, divergences in nongrain silages and straw result in a quadratic impact on milk yield with a similar pattern with a maximum yield around minimum divergence (i.e., 100%). Rossow and Aly (2013) concluded that variability in lignin content of the TMR had the greatest influence on the variability of milk yield; thus, herein the relationship between divergences in straw and nongrain silages and milk yield could also be due to changes in lignin supply. Also, it could be speculated that providing more straw or nongrain silages could facilitate sorting against long particles (Miller-Cushon and DeVries, 2017), which could alter rumen fermentation, DMI, and ultimately milk yield. Similarly, providing less straw or nongrain silages could have negative consequences, not due to sorting but to poor supply of fiber relative to nonfiber carbohydrates.

**Figure 2:**
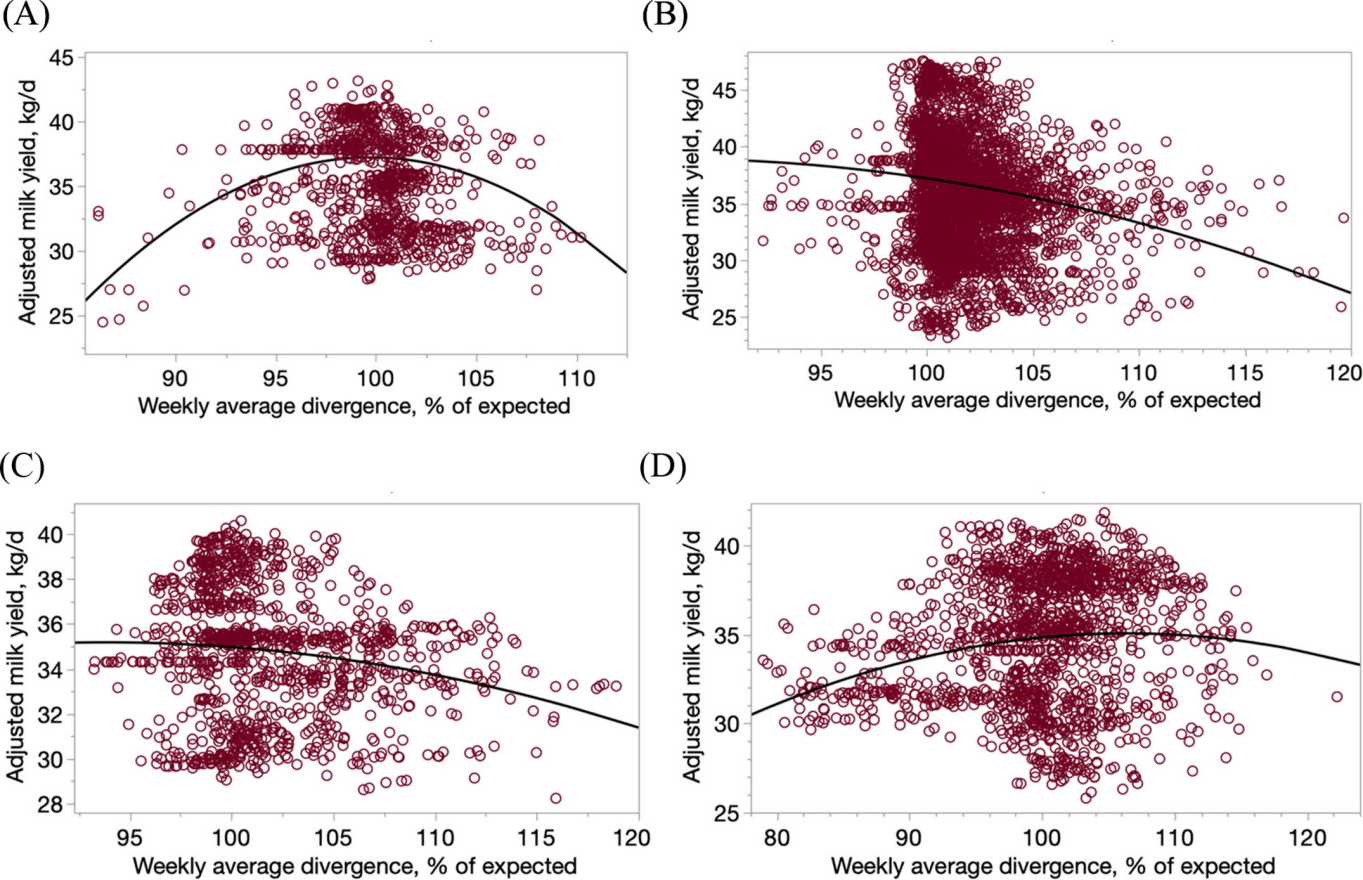
Effect of mixing divergences (% relative to expected amount) in selected ingredient types on milking performance. (A) Nongrain silage (adjusted milk yield, kg/d = −507.27 + 10.952 × weekly average divergence − 0.0551 × weekly average divergence^2^; R^2^ = 0.03, *P* < 0.01). (B) Grain silage (adjusted milk yield, kg/d = −106.97 − 3.142 × weekly average divergence − 0.0170 × weekly average divergence^2^; R^2^ = 0.02, *P* < 0.01). (C) Protein source (adjusted milk yield, kg/d = −62.74 + 1.99 × weekly average divergence − 0.01 × weekly average divergence^2^; R^2^ = 0.03, *P* < 0.01. (D) Straw (adjusted milk yield, kg/d = −55.55 + 1.744 × weekly average divergence − 0.0084 × weekly average divergence^2^; R^2^ = 0.04, *P* < 0.01).

The divergence (data not plotted) between expected and actual amounts for grains (adjusted milk yield, kg/d = −79.97 [±41.1] + 2.447 [±0.799] × weekly average divergence − 0.0128 × weekly average divergence^2^; R^2^ = 0.02, *P* < 0.01), hays (adjusted milk yield, kg/d = −52.67 [±32.5] + 1.730 [±0.625] × weekly average divergence − 0.0083 × weekly average divergence^2^; R^2^ = 0.01, *P* = 0.02), minerals (adjusted milk yield, kg/d = −7,653 [±2,064.8] + 152.75 [±41.15] × weekly average divergence − 0.758 [±0.205] × weekly average divergence^2^; R^2^ = 0.03, *P* < 0.01), and molasses (adjusted milk yield, kg/d = −23.51 [±20.0] + 1.278 [±0.407] × weekly average divergence − 0.0067 [±0.0021] × weekly average divergence^2^; R^2^ = 0.01, *P* < 0.01) was also quadratically (with a concave shape) associated with milking performance. It can be speculated that increases in the proportion of grain in the TMR (due to mixing errors) may cause some rumen upsets and reduce intake and milk yield, and vice versa, not adding sufficient grain in the diet may limit energy availability to sustain milk production. Milk responses to divergences in hay could be attributed to the same reasons discussed previously for straw or nongrain silages. More research would be needed to clarify the reasons behind the association between divergences in minerals and molasses and milk yield. Last, molasses are commonly regarded as highly palatable feedstuff in the field, but Hall and Zanton (2022) reported that as the proportion of molasses in the diet increases, feed intake (and milk yield) decreases, which could partially explain the reduction in milking performance observed herein as divergence in molasses in the TMR increased.

In conclusion, producers mix greater amounts of TMR than they should, based on the number of animals to feed and the ration formulated by the nutritionist. This divergence is mainly caused by excessive amounts of grains, protein sources, hay, and grain silages and deficient amounts of minerals, molasses, straw, and nongrain silages added to the TMR wagon during mixing. This divergence in total amount of feed mixed is quadratically associated with milk yield. Also, divergences in the amounts of individual ingredients are quadratically associated with milk yield.
